# Bergen Barbers: A Student-Run Haircut and Grooming Service to Enhance Empathy, Clinical Comfort, and Patient-Centered Skills in Medical Education

**DOI:** 10.1007/s40670-026-02886-2

**Published:** 2026-09-21

**Authors:** Ivan Loncar, Shivani Srivastava, Sowntharya Ayyappan, Hetal Lad, Amy Gore

**Affiliations:** Rutgers New Jersey Medical School, 185 South Orange Avenue, Newark, NJ 07103 USA

**Keywords:** Service learning, Empathy, Medical education, Patient-centered care, Professional identity

## Abstract

Bergen Barbers is a student-led program at Rutgers New Jersey Medical School, where medical students provide free haircuts and grooming services to hospitalized patients. The program began as a pilot with four student volunteers in its first year and grew to sixteen newly recruited volunteers each academic year. This pilot study was conducted with the Fall 2025 cohort and used pre/post surveys (*n* = 15 pre, *n* = 12 post) to assess changes in empathy, communication, and clinical confidence. Significant improvements were found in five areas: comfort with diverse patients, rapport-building, comfort with physical patient interactions, hospital navigation, and interactions with hospital staff. Students reported high satisfaction and observed visible mood improvements in patients. Bergen Barbers offers a practical model for building humanistic and clinical skills early in medical training.

**Supplementary Information:**

The online version contains supplementary material available at https://doi.org/10.1007/s40670-026-02886-2.

## Background

Pre-clinical medical education presents a persistent challenge: students enter medicine motivated to help people yet spend their first two years largely removed from meaningful patient contact. This period is dominated by didactic coursework, and the gap between classroom learning and real patient interaction is well documented as a source of student frustration and disengagement [1, 2]. Without structured opportunities to practice interpersonal and communication skills early, students may arrive at their clinical rotations underprepared for the human dimensions of patient care.

Research consistently shows that empathy and humanistic care decline as students advance through training [2–4]. This is particularly concerning given that empathy is associated with better patient outcomes, improved diagnostic accuracy, and higher patient satisfaction [5]. The pre-clinical years represent a critical window: students are motivated, reflective, and not yet subject to the time pressures and emotional fatigue of clinical work. Intervening during this period, before negative patterns become established, offers a meaningful opportunity to shape professional identity formation [6, 7].

Service-learning programs that embed students in non-clinical roles with patients have shown promise in addressing this gap. They build communication skills, reduce anxiety around patients, and reinforce the values that attract students to medicine [8, 9]. Additionally, for patients, being in the hospital strips away their daily routine and sense of control [10, 11]. Supportive non-medical interventions may help preserve dignity and normalcy during hospitalization.

Bergen Barbers is a student-founded organization at Rutgers NJMS in which first and second-year medical students provide complimentary haircuts and grooming services (e.g., beard trims, hair combing) to inpatients at University Hospital, under faculty oversight and direct training and supervision of experienced student Executive board (E-board) members. Nurses on the hospital floors directly request the group to visit patients who either request services themselves or have been in the hospital for a long time. At the start of each academic year, a new cohort of students joins the club and may continue contributing throughout medical school. As new students join each year, the program continues to grow and has become an ongoing part of the pre-clinical experience rather than a one-time interaction. The program was designed to give pre-clinical students structured, low-stakes exposure to real patients at a stage when formal clinical contact is limited. By engaging patients through grooming rather than clinical tasks, students practice rapport-building, compassionate communication, and dignified physical interaction without the pressure of clinical responsibility.

This pilot study evaluated whether participation in Bergen Barbers was associated with measurable changes in students’ self-reported empathy, communication skills, and clinical confidence over one academic year.

## Activity

All newly recruited Bergen Barbers members in Fall 2025 were invited to participate in a quantitative pre/post survey study (IRB Pro2025000628, Rutgers University, Expedited). Participants provided informed consent electronically prior to their first program event. An anonymous pre-survey was administered before students’ first Bergen Barbers session, with a corresponding anonymous post-survey administered at the end of the academic year. Bergen Barbers began as a pilot with four student volunteers in its first academic year in Spring 2024. This program gained sixteen new members each academic year (Fall 2024 and Fall 2025). The surveyed cohort reflects the sixteen recruited new volunteers who joined in Fall 2025, the program’s third academic year, and does not include the four pilot academic year volunteers or the sixteen recruited in the second academic year.

Survey instruments included Likert-scale items (1–5) assessing self-reported empathy, self-reported communication ability, comfort in clinical environments, confidence in patient interactions, and familiarity with hospital settings. The post-survey additionally included program-specific items regarding students’ perceptions of patient benefit and personal development. Surveys were hosted on the university’s secure Microsoft Forms platform, and no identifying information was collected.

Bergen Barbers’ events occurred throughout the academic year and consisted of 1 to 2-hour sessions every other week in which students provided haircuts and shaves to hospitalized patients while practicing patient-centered communication. Before independently providing services, new members completed hands-on haircutting and shaving training with an experienced E-board member who had at least one year of prior experience providing haircuts through the program. After this, training included the E-board member observing the new member’s initial patient interactions, providing real-time feedback, and demonstrating proper technique [12]. All technical competency was developed through this structured peer-training model. Nursing staff were present on the hospital floor throughout every session, with a staff member either in the room or immediately available on the hospital floor at all times. Volunteers were not required to hold cosmetology licensure or prior grooming experience. Patients were informed of the program’s voluntary, non-professional nature prior to each session and no separate written waiver specific to the grooming service was obtained.

Each participant’s total survey time commitment was approximately 20 min across both surveys. An unpaired design was used because surveys were fully anonymous and no identifiers were collected. Future iterations will assign anonymous participant codes at orientation to enable within-person comparison and a paired study design. Due to an unpaired study design, independent samples t-tests were used to compare group means. Data were analyzed using R Version 4.5.3. Statistical significance was set at *p* < 0.05. Effect sizes were calculated using Cohen’s d.

## Results and Discussion

**Fig. 1:**
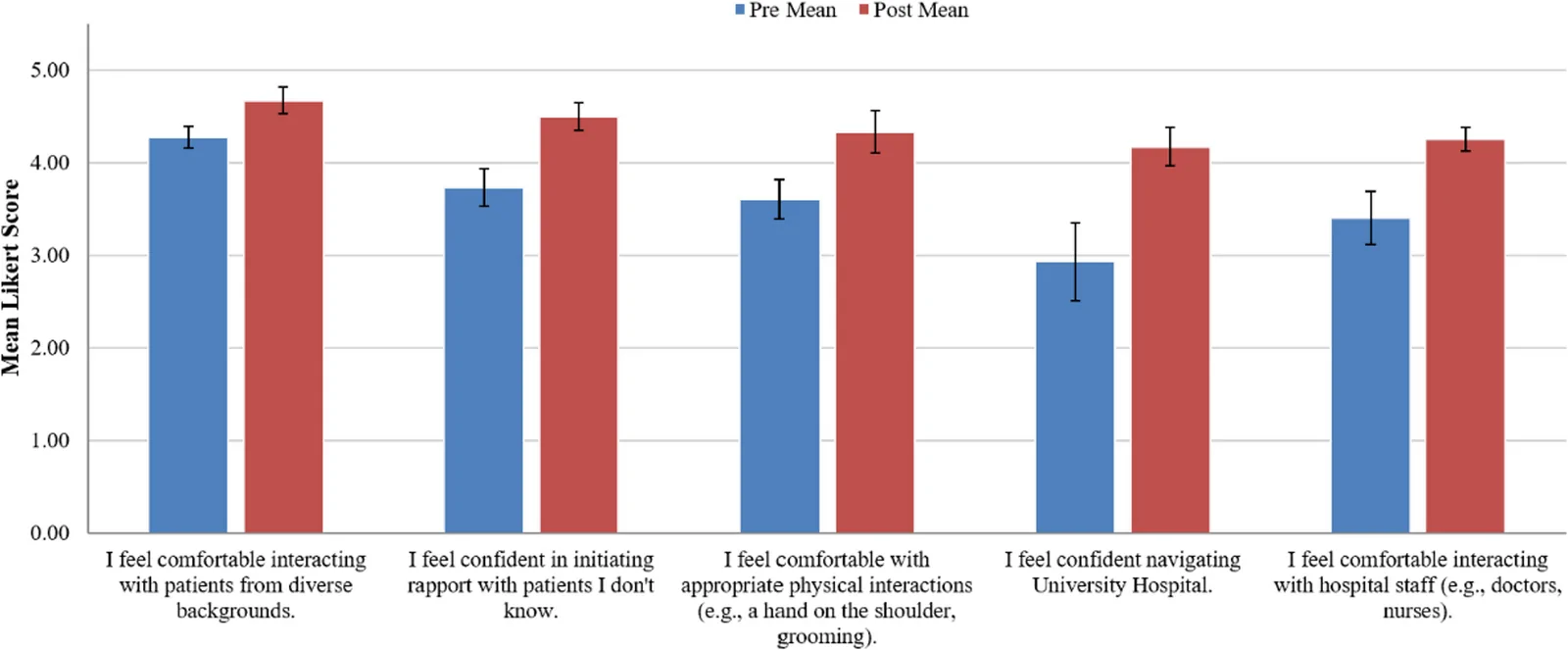
Mean pre- and post-program likert scores (± standard error) for the five survey items that showed significant improvement following the program. *P* < 0.05 for all comparisons

Fifteen students completed the pre-survey and twelve completed the post-survey. Five items showed statistically significant improvement from pre- to post-program, all with large effect sizes (Fig. 1,Table 1).

**Table 1:** Pre- and post-program survey responses for selected items (*n* = 15 pre, *n* = 12 post). Mean Likert scores (1 = strongly disagree, 5 = strongly agree)

Survey measure	Pre mean (SD)	Post mean (SD)	*p*	Cohen’s d
Empathy and Patient Interaction Measures				
Empathy is an essential skill in medicine.	4.93 (0.26)	4.92 (0.29)	0.88	-0.06
I know how to respond empathetically when a patient expresses frustration, sadness, or fear.	4.07 (0.46)	4.08 (0.51)	0.93	0.03
I feel comfortable being in emotionally sensitive patient encounters.	4.13 (0.52)	4.25 (0.87)	0.69	0.17
I feel comfortable interacting with patients from diverse backgrounds.	4.27 (0.46)	4.67 (0.49)	0.04*	0.85
I feel at ease entering a patient’s room and introducing myself.	4.33 (0.49)	4.58 (0.51)	0.21	0.50
I feel confident in initiating rapport with patients I don’t know.	3.73 (0.80)	4.50 (0.52)	0.01*	1.11
I feel comfortable with appropriate physical interactions (e.g., a hand on the shoulder, grooming).	3.60 (0.83)	4.33 (0.78)	0.03*	0.91
Clinical Environment and Team Comfort Measures				
I feel confident working in a team in a patient care setting.	4.40 (0.63)	4.75 (0.45)	0.11	0.62
I feel comfortable learning in hospital settings.	4.20 (0.77)	4.50 (0.67)	0.29	0.41
I feel confident navigating University Hospital.	2.93 (1.62)	4.17 (0.72)	0.02*	0.94
I feel comfortable interacting with hospital staff (e.g., doctors, nurses).	3.40 (1.12)	4.25 (0.45)	0.01*	0.95
Beliefs About Patient Dignity and Non-Medical Care				
Physical appearance can affect a patient’s emotional and psychological well-being.	4.67 (0.49)	4.75 (0.45)	0.65	0.18
I believe non-medical interactions (e.g., grooming, talking) can improve the overall hospital experience for patients.	4.67 (0.49)	4.83 (0.39)	0.33	0.37
I think hospitals should provide more services that address the emotional and dignity-related needs of patients.	4.67 (0.49)	4.75 (0.45)	0.65	0.18
I can learn clinically relevant information about medical conditions and treatments while supporting patients in a non-medical capacity.	4.67 (0.49)	4.50 (0.67)	0.48	-0.29

*p < 0.05. *M* mean, *SD* standard deviation *d* Cohen’s d effect size.

The largest mean increase was in confidence navigating University Hospital (pre M = 2.93, post M = 4.17; *p* = 0.02, d = 0.94), a competency rarely addressed in pre-clinical curricula yet consistently identified as a barrier when clinical rotations begin [1]. Significant improvements were also found in comfort interacting with patients from diverse backgrounds (*p* = 0.04, d = 0.85), confidence initiating rapport with unfamiliar patients (*p* = 0.01, d = 1.11), comfort with physical patient interactions such as grooming (*p* = 0.03, d = 0.91), and comfort interacting with hospital staff (*p* = 0.01, d = 0.95). Several additional items trended in a positive direction but did not reach significance, including confidence working in a team (d = 0.62) and ease entering a patient’s room (d = 0.50). The large effect sizes in these underpowered comparisons suggest they warrant attention in future paired designs with greater sample sizes.

Empathy and communication items did not show significant change, likely due to ceiling effects: pre-survey means for items such as “empathy is essential in medicine” (M = 4.93) and “non-medical interactions improve patient experience” (M = 4.67) were already near the scale maximum, leaving limited room for measurable growth. This pattern is consistent with prior research showing that near-universal endorsement of humanistic values at baseline can limit the detectability of change in empathy-focused interventions [4]. Also, students who chose to join Bergen Barbers may already value empathy highly, contributing to the ceiling effects.

Post-program data (Table 2) revealed uniformly high satisfaction as 100% of respondents found Bergen Barbers enjoyable and rewarding (M = 4.92), and students strongly endorsed visible mood improvement in patients after grooming (M = 4.92). Students also reported that participation heightened their awareness of actions that contribute to patient dignity (M = 4.83) and strengthened their sense of belonging within the medical school (M = 4.58). Notably, students did not strongly endorse needing additional hands-on practice prior to volunteering (M = 3.75) or a dedicated empathy skills session (M = 3.92), suggesting that the program’s naturalistic, low-stakes format may itself serve as a sufficient stage for pre-clinical patient contact. Moreover, the findings on patient mood are consistent with evidence that humanizing non-clinical interventions can meaningfully improve the inpatient experience [11, 13, 14].

**Table 2:** Post-survey responses among Bergen Barbers participants (*n* = 12). Mean Likert scores (1 = strongly disagree, 5 = strongly agree) for post-program survey items

Post-survey measures only	Post mean (SD)
Providing grooming services has made me more aware of the small acts that contribute to patient dignity and quality of life.	4.83 (0.39)
I notice a visible improvement in patients’ mood after receiving a haircut.	4.92 (0.29)
I find participating in Bergen Barbers to be an enjoyable and rewarding experience.	4.92 (0.29)
I look forward to Bergen Barbers events as a break from the academic pressures of medical school.	4.67 (0.49)
The club has strengthened my sense of community and belonging within the medical school.	4.58 (0.51)
I would benefit from a session on empathy and interpersonal skills.	3.92 (1.00)
Bergen Barbers provided me with sufficient volunteering opportunities.	4.75 (0.45)
I receive sufficient support from club leadership (training, supplies, guidance).	4.42 (0.67)
I would benefit from additional hands-on practice sessions before volunteering with patients.	3.75 (0.97)
I would recommend Bergen Barbers to other medical students.	4.83 (0.39)

These findings align with literature on service-learning in health professions education, which shows that structured non-clinical patient contact can reinforce humanistic values and support professional identity formation early in training [8, 15]. The significant gains in hospital navigation and comfort with staff interactions are particularly relevant, as these practical competencies are rarely addressed in pre-clinical curricula yet represent real barriers to student confidence when clinical rotations begin [1]. The large effect sizes across all five significant items suggest that even brief, recurring non-clinical patient contact can produce meaningful clinical development in pre-clinical students.

Several limitations should be noted. The unpaired design precludes within-person comparison and reduces statistical power. The small sample size limits generalizability, and self-report measures are subject to social desirability bias. Because not all eligible participants completed both surveys, nonresponse bias is possible if students who chose not to participate differed systematically in their experiences or perceptions of the program. Additionally, the number of sessions and patient encounters completed varied across participants and was not tracked in this pilot, so individual differences in exposure may have influenced self-reported outcomes. However, all students were expected to participate for a minimum of 2 h per month. The absence of a control group means improvements cannot be attributed exclusively to Bergen Barbers participation. Given the exploratory nature of this pilot, p-values were not adjusted for multiple comparisons, and findings should therefore be interpreted as preliminary. Future iterations will employ a paired design with anonymous identifiers assigned as orientation, along with longitudinal follow-up into clinical years to assess whether gains are sustained. As Bergen Barbers continues to grow annually, successive cohorts offer an opportunity to strengthen the evidence base and track professional identity development over time. Future work will also examine the patient perspective directly, including measures of mood, dignity, and satisfaction with the inpatient experience to complement the student-reported outcomes presented here.

## Conclusion

Bergen Barbers produced meaningful improvements in hospital navigation, rapport-building, comfort with diverse patients, physical patient interactions and staff communication among pre-clinical medical students over one academic year. Students perceived visible benefits to patient mood and dignity and reported strong satisfaction with the program. As a student-founded, faculty-supervised program requiring no dedicated curriculum time, Bergen Barbers offers a practical model for incorporating structured service learning and professional identity formation into pre-clinical medical education.

## Supplementary Information

Below is the link to the electronic supplementary material.

Supplementary Material 1

## References

[1] Surmon L, Bialocerkowski A, Hu W. Perceptions of preparedness for the first medical clerkship: a systematic review and synthesis. BMC Med Educ. 2016;16:89. 10.1186/s12909-016-0615-3. PMC4788861 26968816

[2] Hojat M, Vergare MJ, Maxwell K, Brainard G, Herrine SK, Isenberg GA, Veloski J, Gonnella JS. The devil is in the third year: a longitudinal study of erosion of empathy in medical school. Acad medicine: J Association Am Med Colleges. 2009;84(9):1182–91. 10.1097/ACM.0b013e3181b17e55. 19707055

[3] Ebm C, Sarti R, Panico P, Pagliotta M, Vinci V, Oldani S. Enhancing compassion in medical education - a comparative study of the efficacy of clinical clerkships versus simulation-based training methodologies. BMC Med Educ. 2025;25(1):181. 10.1186/s12909-025-06687-w. PMC11796239 39905468

[4] Boshra M, Lee A, Kim I, Malek-Adamian E, Yau M, LaDonna KA. When patients teach students empathy: a systematic review of interventions for promoting medical student empathy. Can Med Educ J. 2022;13(6):46–56. 10.36834/cmej.73058. PMC9684039 36440084

[5] Derksen F, Bensing J, Lagro-Janssen A. Effectiveness of empathy in general practice: a systematic review. Br J Gen practice: J Royal Coll Gen Practitioners. 2013;63(606):e76–84. 10.3399/bjgp13X660814. PMC3529296 23336477

[6] Holden MD, Buck E, Luk J, Ambriz F, Boisaubin EV, Clark MA, Mihalic AP, Sadler JZ, Sapire KJ, Spike JP, Vince A, Dalrymple JL. Professional identity formation: creating a longitudinal framework through TIME (Transformation in Medical Education). Acad medicine: J Association Am Med Colleges. 2015;90(6):761–7. 10.1097/ACM.0000000000000719. 25853688

[7] Thistlethwaite JE, Bartle E, Chong AA, Dick ML, King D, Mahoney S, Papinczak T, Tucker G. A review of longitudinal community and hospital placements in medical education. BEME Guide 26 Med teacher. 2013;35(8):e1340–64. 10.3109/0142159X.2013.806981. 23848374

[8] Yang YS, Liu PC, Lin YK, Lin CD, Chen DY, Lin BY. Medical students’ preclinical service-learning experience and its effects on empathy in clinical training. BMC Med Educ. 2021;21(1):301. 10.1186/s12909-021-02739-z. PMC8157642 34039327

[9] Tang JW, Kostas T, Verma A, Press VG, Kushner J, Gier N, Wiklund LO, Arora VM, Farnan J, Meltzer DO, Ley AG, Rawson L, Redley H, B., Kerr D. (2022). A systematic review of patient-reported dignity and dignified care during acute hospital admission. Journal of advanced nursing, 78(11), 3540–3558. 10.1111/jan.15370 PMC9795980 35841334

[10] Baillie L. Patient dignity in an acute hospital setting: a case study. Int J Nurs Stud. 2009;46(1):23–36. 10.1016/j.ijnurstu.2008.08.003. 18790477

[11] Bohrer MB, Rodgers DL. Improving confidence in performing clinical procedures through peer-driven training sessions for preclinical medical students. MedEdPORTAL. 2025;21:11542. 10.15766/mep_2374-8265.11542. PMC12361509 40836949

[12] Mendelson A, Bandfield B, Hevezi J, Gable J, Davidson JE, Buckholz G. Using photographs to bring dignity to patients and help clinicians find meaning and joy in work. J Palliat Med. 2023;26(2):165–74. 10.1089/jpm.2022.0161. PMC9894591 35997617

[13] Richard A, Harbeck N, Wuerstlein R, Wilhelm FH. Recover your smile: effects of a beauty care intervention on depressive symptoms, quality of life, and self-esteem in patients with early breast cancer. Psycho-oncology. 2019;28(2):401–7. 10.1002/pon.4957. PMC6590176 30511433

[14] Kovaric K, Luk J. Impact of a service learning curriculum that emphasizes bidirectional learning on medical students’ professional identity formation. Med Sci Educ. 2024;35(1):371–9. 10.1007/s40670-024-02202-w. PMC11933504 40144070

[15] Lu WH, Pang L, Strano-Paul L. The impact of COVID-19 service learning on medical student professional identity formation. J Med Educ Curric Dev. 2024;11:23821205241262686. 10.1177/23821205241262686. PMC11425726 39328821

