# Supplementary material for: Bergen Barbers: A Student-Run Haircut and Grooming Service to Enhance Empathy, Clinical Comfort, and Patient-Centered Skills in Medical Education
Source: Med Sci Educ. 2026 Sep 21;36(5):2151–5. doi: 10.1007/s40670-026-02886-2 (PMC13723476; doi:10.1007/s40670-026-02886-2)
Supplement: Supplementary file 1 [file 40670_2026_2886_MOESM1_ESM.docx]

**Response to Reviewers**

Bergen Barbers: A Student-Run Haircut and Grooming Service to Enhance Empathy, Clinical Comfort, and Patient-Centered Skills in Medical Education | MDSE-D-26-00998

| **#** | **Reviewer Comment** | **Author Response** |
| --- | --- | --- |
| **Editor** | Reviewers found the pilot project from Loncar et. al fitting as a commentary in Medical Science Educator. Ahead of consideration, please address all items highlighted by the reviewers. In particular provide more recent citations relevant to the work, improve the layout of the data, and provide a formal version for submission. | We thank the Editor for the positive assessment of our work. We have addressed each reviewer comment below, updated the reference list with more recent citations, added Figure 1, and are submitting a clean, formally formatted version of the manuscript. |
| **Review 1** | | |
| 1.1 | Overall, this short communication manuscript represents a rather innovative pilot study to enhance patient interaction skills in medical students. The manuscript has several strengths, including significant results, but it is not complete. There are several comments (in the form of "track changes") throughout the manuscript that need to be addressed and/or resolved by the authors. | We thank the reviewer for this encouraging assessment. We have resolved all embedded track-changes comments and uploaded a clean formal version of the manuscript. |
| 1.2 | There is a comment on page 5 of the pdf that says, "This isn't necessarily true?" regarding the statement that participants were "under hospital staff supervision" (line 37). The authors need to resolve this comment and may need to revise the manuscript if study participants were not supervised by hospital staff. | We have revised this statement for accuracy. The ambiguous phrase "under hospital staff supervision" has been removed. |
| 1.3 | The manuscript reports significant results overall, although, again, there are unresolved comments that make interpretation of the results uncertain. | Same as Comment 1.1, resolved. |
| 1.4 | Conclusion and summary are appropriate for a short communication manuscript. | No changes made. |
| 1.5 | Most of the references are over 10 years old, they should be updated. | We have added seven new citations published 2019-2026 and removed three of the most dated references. |
| 1.6 | Tables are appropriate. | Kept tables and added Figure 1 showing significant findings. |
| **Reviewer 2** | | |
| 2.1 | I appreciate a service-learning program that aims to increase student engagement and provide opportunities for interaction with patients. I do agree that students become frustrated during their preclinical years because they feel so far removed from all the reasons that they wanted to become a doctor. I also really love the opportunity for students to get to interact with REAL patients in non-clinical ways instead of only simulated patients in clinical ways during their preclinical years. I think simulations are very important, but there is still an extra layer of nerves for students to talk with real patients knowing they are not in a simulation environment. I agree many of the bedside skills needed could be developed and improved upon during non-clinical tasks. | Thank you! |
| 2.2 | It appears that the wrong version of the manuscript was uploaded to the review site as comments from the authors to one another during the review process are visible on the uploaded PDF. | We apologize for this oversight. A clean, final version of the manuscript with no embedded author comments is included with this resubmission. |
| 2.3 | The sample size numbers are a little unclear to me. The program sees a growth of 16 new members "each year," but the program is also only in its second year. Would it be more accurate to say the program involved XX students in its first year and grew to XX members in its second year? Also, does Fall 2025 when the survey was administered represent the first year of Bergen Barbers or the second year? If it is the second year, I would assume there were something like 30 students total in the program, but the survey numbers would seem to fit better with the first year.... | We have revised the Abstract and Activity section to clarify program timeline and membership. |
| 2.4 | There were 15 pre-surveys completed and 12 post-surveys completed, but how many members were there total in Bergen Barbers at the time? Does this sample represent the entire program as a whole? Or could other students potentially did not feel the program was beneficial and thus did not want to fill out the pre/post surveys? | Added sentence in limitation paragraph: “Because not all eligible participants completed both surveys, nonresponse bias is possible if students who chose not to participate differed systematically in their experiences or perceptions of the program” |
| 2.5 | Were all students involved to the same extent? In other words, did all students participate in approximately the same number of haircuts? Or could experiences be different because some students saw X number of patients during the year while others saw 2X or 3X patients? | We have added a limitation noting that individual participation (number of haircuts/patient encounters) was not uniform across students and was not tracked in this pilot, which may have influenced individual survey responses. Students were expected to participate for a minimum of 2 hours per month. |
| 2.6 | I agree some of the results did not show significant change because of ceiling effects. Students in medicine likely already value concepts of empathy, and then on top of that, students that choose to be involved in the barber program would most likely be some of the most empathetic of all the students. | Added sentence in Discussion: “Also, students who chose to join Bergen Barbers may already value empathy highly, contributing to the ceiling effects” |
| 2.7 | In future iterations of the survey, you could add a question about the Bergen Barbers being an outlet to increase patient interactions in the preclinical years. The question could try to ask something along the lines of although the encounter is not clinical, does it provide meaningful experience/education regarding the patient/doctor interaction? Is it a meaningful experience that helps ease student frustration at the lack of patient interaction? One would assume these answers would be yes, but I felt this was a strong point made in your background and it might be nice to "tie it together" with the presented results. | Thank you for your suggestion! We will consider this for our next iteration. |
| 2.8 | The conclusions and limitations highlighted seem appropriate for the study. | No changes. |
| 2.9 | For Table 1 I would include the 1=strongly disagree and 5= strongly agree statement that is included with table 2. | This has been added. |
| **Reviewer 3** | | |
| 3.1 | The Bergen Barbers program as described is a great way to introduce students to patients in a "low-stakes format (as the authors express) but with personal interactions. Many students who apply to medical school have next to zero experience with "customer service" which I believe is a real gap in life's experience. Patients are customers, like it or not. Thus, overall I applaud the authors efforts in getting the word out about this unique program which seems to benefit the students and the patients, as their data show. What a grand idea! | Thank you! |
| 3.2 | As the spouse of a licensed cosmetologist, I do need to ask who was "allowed" to perform haircuts and "grooming" (which should be defined)? Did volunteers have to be cosmetologists or at least have documented skills? Receiving a bad haircut can ruin someone's day! Did the patients sign a waiver releasing the student(s) from being blamed for a bad haircut? On this note, the title states a "...student led grooming program..." which is a bit of an odd title because of the various contexts of such a phrase. | Added more details to Activity section on services provided and training protocol. We have also revised the title to avoid ambiguity. |
| 3.3 | Simple Likert scale questionnaire seems appropriate, and that this was a pilot study (as a short communication) requiring just over a dozen student participants is adequate. | No changes made. |
| 3.4 | My only feedback is that I'd rather see graphs than a big table of data. Text describing the data aptly captured the details. | Kept tables and added Figure 1 showing significant findings. (See response 1.6) |
| 3.5 | "These findings align with literature on service-learning in health professions education..." : wonderful summary line. Discussion and conclusion were clear and succinct. Nicely done. | No changes made. |
| 3.6 | A little dated, only a couple articles from 2022 and beyond. There must be more recent publications to cite? | Done. See response 1.5 above. |
| 3.7 | I'd turn the tables into graphs. Too many numbers. | Kept tables and added Figure 1 showing significant findings. (See response 1.6 and 3.4) |
